# Retraction Note: LncRNA CCAT1 facilitates the progression of gastric cancer via PTBP1-mediated glycolysis enhancement

**DOI:** 10.1186/s13046-026-03737-z

**Published:** 2026-05-16

**Authors:** Cong Zhang, Huixia Wang, Qingwei Liu, Suli Dai, Guo Tian, Xintong Wei, Xiaoya Li, Lianmei Zhao, Baoen Shan

**Affiliations:** 1 Research Center, the Fourth Hospital of Hebei Medical University, Jiankang Road 12, Shijiazhuang, Hebei 050011 China; 2 Key Laboratory of Tumor Gene Diagnosis, Prevention and Therapy; Clinical Oncology Research Center, Shijiazhuang, Hebei 050001 China; 3 Third Department of Surgery, the Fourth Hospital of Hebei Medical University, Shijiazhuang, Hebei 050011 China; 4 Medical Records Department, the Fourth Hospital of Hebei Medical University, Shijiazhuang, Hebei 050011 China

**Retraction Note: J Exp & Clin Cancer Res 42, 246 (2023)**

**https://doi.org/10.1186/s13046-023-02827-6**

The Editor-in-Chief has retracted this article. After publication, concerns were raised regarding data and image irregularities and animal ethics approval. Specifically, Fig. 3F, PKM1 (siNC) and Fig. 3F, PKM1 (CCAT1) appear to be highly similar. Upon request, the authors provided the original data, however, the blot provided for Fig. 3F, PKM1 (siNC) appears to show a discrepant result to the one in the article.

Concerning the animal in vivo studies, it appears that the research was not conducted in compliance with international best practice standards as the ethics approval document did not match with the experiments carried out in this study.

In addition, MGC-803, SGC-7901 and BGC-823 cell lines have been reported to be contaminated with HeLa and are therefore unsuitable for gastric cancer research [1–4].

Therefore, the Editor-in-Chief no longer has confidence in the presented data.

Lianmei Zhao agrees with this retraction.

Cong Zhang, Huixia Wang, Suli Dai, Xintong Wei and Xiaoya Li did not explicitly state whether or not they agree with this retraction.

Qingwei Liu, Guo Tian and Baoen Shan did not respond to correspondence from the Publisher about this retraction.

## References

[1] Ye F, Chen C, Qin J, Liu J, Zheng C. Genetic profiling reveals an alarming rate of cross-contamination among human cell lines used in China. FASEB J. 2015;29(10):4268–72. 26116706 10.1096/fj.14-266718

[2] Bian X, Yang Z, Feng H, et al. A combination of species identification and STR profiling identifies cross-contaminated cells from 482 human tumor cell lines. Sci Rep. 2017;7:9774. 10.1038/s41598-017-09660-w. 28851942 10.1038/s41598-017-09660-w PMC5575032

[3] Cao F, Sun H, Yang Z, Bai Y, Hu X, Hou Y, et al. Multiple approaches revealed MGc80‐3 as a somatic hybrid with HeLa cells rather than a gastric cancer cell line. Int J Cancer. 2024;154(1):155–68. 37543987 10.1002/ijc.34677

[4] Yang M, He J, Xia S, et al. Investigation of the mixed origins of the MGC-803 cell line reveals that it is a hybrid cell line derived from HeLa. Hum Cell. 2024;37:560–6. 10.1007/s13577-023-01011-4. 38079103 10.1007/s13577-023-01011-4

